# Editorial: SLC6A1: the past, present and future

**DOI:** 10.3389/fnins.2023.1289821

**Published:** 2023-10-12

**Authors:** Katrine M. Johannesen, Eduardo Pérez-Palma, Guido Rubboli

**Affiliations:** ^1^Department of Epilepsy Genetics and Personalized Treatment, The Danish Epilpesy Centre, Dianalund, Denmark; ^2^Department of Genetics, Rigshospitalet, University Hospital of Copenhagen, Copenhagen, Denmark; ^3^Facultad de Medicina Clínica Alemana, Centro de Genética y Genómica, Universidad del Desarrollo, Santiago, Chile; ^4^Institute of Clinical Medicine, University of Copenhagen, Copenhagen, Denmark

**Keywords:** SLC6A1, epilepsy, epilepsy genetics, neurodevelopmental disorders, genetics

Within the past years, our knowledge on *SLC6A1*-related neurodevelopmental disorders (*SLC6A1*-NDDs) have increased tremendously. However, there are still many aspects of this disorder that needs further elucidating. As we move closer to targeted treatment in *SLC6A1*-NDDs, we need a clear understanding of the disorder including the phenotypic range and natural history. While large cohort studies might not be available yet for *SLC6A1*-NDD, there are many international efforts underway to improve the available knowledge and raise awareness. *SLC6A1*-NDDs are among the top ten most common monogenic causes in both autism and epilepsy, and thus one of the more common among the rare. Advancements made in *SLC6A1*-NDDs might not only improve treatment in patients with this specific disorder, but may also affect treatment options in other rare DEEs.

To further increase our understanding, we need both clinical and molecular studies involving patients but also experimental models, exemplified by Kasture et al. who used the drosophila melanogaster fly model to evaluate the trafficking effect of several *SLC6A1* variants. Several of these variants showed reduced trafficking being trapped in the ER. Interestingly, the study showed that this trafficking defect might be rescuable by pharmacochaperoning, adding to the list of possible treatment targets in *SLC6A1*-NDDs.

The “typical” *SLC6A1*-NDD phenotype is characterized by epilepsy, most commonly epilepsy with myoclonic atonic seizures (EMAS) starting in infancy/early childhood and accompanied by intellectual disability, behavioral disorders and stereotypies. As with many other disorders, *SLC6A1*-NDDs cannot be encompassed within the EMAS diagnosis. This is illustrated by Kalvakuntla et al., who provided further description of patients that experience periods of developmental regression, especially after seizure onset. A clear correlation between those who had regression and those who did not was not apparent, but hints toward a higher prevalence of autism and severe language delay in those with regression was observed. In the study by Caputo et al., two patients display a phenotype of absence seizures but not EMAS. This confirms previous reports that found that childhood onset absence epilepsy is also a part of the phenotypic spectrum of *SLC6A1*-NDDs.

Finally, Kassabian et al. describe a cohort of families with intrafamilial variability, where not all variant carriers display a “typical” *SLC6A1*-NDD phenotype. Instead parents and in some families even grandparents present with milder or incomplete forms of the disorder, suggesting that phenotypic spectrum may be even wider and/or that the clinical presentation might be modulated by additional rare or common genetic factors.

Our current knowledge in *SLC6A-*NDDs mainly arise from pediatric cohorts and case reports in children, as also displayed by the majority of papers presented in this Research Topic. However, as the availability and use of genetic tests have expanded, we now also have a small population of adults with *SLC6A1*-NDDs. Johannesen et al. present a phenotype in adults similar to that reported in children, albeit maybe more severe with refractory seizures in a number of individuals, highlighting the need for better treatment options in *SLC6A1*-NDDs to change the outlook for these patients.

In rare diseases like *SLC6A1*-NDD research will often focus on the disease characteristics that are the most obvious to clinicians, while sometimes failing to pay attention to what is important to families and what are the major issues impairing quality of life in these families. In the study by Goodspeed et al. this is pointed out via analysis of social media interactions between families, where not only epilepsy and autism were prominent issues discussed among parents, but also other topics such as behavioral disorders. The same was noticed when interviewing key opinion leaders within the *SLC6A1* community, who noted stereotypies and developmental regression (as described above) to be understudied areas.

Further, Trivisano et al. stress the importance of neuropsychological evaluation, as at least one-third of patients have behavioral disorders. Additonally, in line with Kalvakuntla et al., they also found that cognitive impairment could worsen over time, highlighting the usefulness of continued neuropsychological evaluation in patients with *SLC6A1*-NDDs. Individual assessments could then lead to tailored rehabilitation programs for these patients.

For future research in *SLC6A1*-NDD it will be important to listen to patients and families, as they will provide the full characterization of the disorder as well as highlight which areas are the most obstructive for quality of life. The outcome of such studies will be relevant as we approach precision medicine in *SLC6A1*-NDDs. With both anti-sense oligonucleotides (ASO)-related therapies and gene therapy on the horizon for *SLC6A1*-NDDs it will remain important that we know which clinical characteristics to evaluate and which outcomes we should aim at, when assessing these new treatments.

## Author contributions

KJ: Writing—original draft, Writing—review and editing. EP-P: Writing—review and editing. GR: Writing—review and editing.

